# Influence of Example

**Published:** 1864-07

**Authors:** W. H. Atkinson


### INFLUENCE OF EXAMPLE.

Read before the Society of Dental Surgery of the city of New York April 6th, 1864.

BY W. H. ATKINSON.

Influence of example is so potent upon nine in ten of the workers upon the stage of human activities, that we can hardly over estimate the importance of correct deportment in all that we do.

Although it is no great compliment to a body of geniuses, as I have often denominated our body to be, nevertheless the disposition to follow in easy, well-beaten paths is infinitely more prevalent among us than the necessary boldness, courage and completeness of purpose to have straight, plain paths, in which to walk, even if they had to be slowly cut through the dense forest of ignorance of the goodness of use, at the sacrifice of the life of many a noble pioneer.

He who will not take the trouble to *think* can never arrive at the independence of *knowing.* Just so in all our attempts to accomplish the works of redemption which engage our heads and hands at our daily toil. If we have no originality of method, we shall often be baffled in our purposes, and have to write down the great majority of our attempts as "*failures;*" simply because we can not remember the detail of formula we have read or heard which appertains to the case. And yet we should be careful that we do not entrap ourselves into saying that we *know*, when we only *think.*

He who *knows* can demonstrate in works or words all the matters reduced to *knowledge.* What *has been* and what *is* the example of those who are acknowledged to be the best dentists? Until very lately it has been such as to lead the people and the pupil of dentistry to believe that skill and comparative superiority of manipulative ability was a plant of rapid growth and easy of culture.

This pernicious example hangs like an incubus upon the neck of the profession and people to such a degree that a very small minority of the former even now dare to attempt any thing at all above the lowest ability, and only a few of the more intelligent of the latter hope to attain permanent or considerable advantage by the employment of a dentist.

One kind of boldness, amounting to recklessness, is sufficiently abundant among us, viz.: That of dubbing every thing in advance of low states of apprehension and ability, as "humbug," "pretension," and "the pet follies of enthusiastic hair brains," &c., &c.

If all such objectors would give but a tithe of the pathos and persistent energy with which they decry what they do not understand, to the earnest, honest investigation, "whether these things be so," an example would be set by them that would not only redeem them from error themselves, but also convert them from setters of *bad* to advocates and practitioners of *good* example!

He who has sufficient perception of right relations and rectitude of character to desire that *his* example shall be good at all times and upon all who may be influenced by it, is in no specially enviable predicament, in view of the multitudinous chances for misapprehension, misinterpretation, misrepresentation and directly malicious perversion of even his best attempts to do no harm and all the good he can. And it often turns out that those well endowed and well disposed are discouraged from exposing themselves to criticism by unkind and unfeeling, coarse characters, by opening out the treasure house of their attainments, which, were it but once fairly begun, would go far to disarm even diabolism itself of all its feared criticism, and turn it to admiration or the expression of interested desire for further entertainment of the same sort. Is, then, the reticence of the regular attendants upon the meetings of this body a good or bad example?

The character of this example may be easily determined by supposing that it were generally followed. It is plain that were it practiced, we would soon have no stimulus to bring us within the charmed circle of co-fraternal magnetisms, and thus we should lose all the polarizing influence of mutual example and excitement to higher and completer works of head, heart and hand, by shutting off the potent exponent of these in the burning words that issue from the mouth that is opened wide to bless us with the pronouncement of the highest attainments of our highest states.

There is another *bad* example, or rather habit, into which self-made operators are liable to fall, and alas! into which some of the brightest examples of ability among us have fallen, if repeated report is to be credited; viz.:

First. The thought that they are really in the advance of all their brethren; and, second, the claim made to the people as incentive for them to be very careful to put their dental organs under the only competent skill—the possessor of which modesty forbids me to name.

Now what should be done in such circumstances, by him who honestly regards himself as head and shoulders above all his fellows? I answer, let him sedulously compare his attainments with the best examples of the fraternity, and then if it proves that his claim is just, let him next impart so far as he is able the advanced attainment to the most worthy in the profession, and thus enlarge himself in usefulness by so many as he adds to the corps of first-class operators, which is certain to bring him greater returns in reputation, patronage, and above all, the blessed consciousness of having been of *use*, than a life-long exclusiveness and attestation of detestation of every other man in his line as unfit to be named as his equal.

Progress in professional attainment is very much like progress in religion. That which is the result of deep conviction and rapid conversion is apt to be generous and boisterous. While that quiet integral progress of which the subject is scarcely conscious himself, until he compares distant periods, is modest, vacillating and uncertain, till long reflection and comparison enables him to say: "The things which I once loved I now hate, and the things which I once hated, I now love;" he comes to the deliberate conclusion that he is really a child of God; but he is in no haste to proclaim that which has become so commonplace to him, and he is quite content to inherit the good things of the kingdom without disturbing his equanimity to have others made partakers of the rich blessings which he feels himself entitled to somehow, but just why, he has no definite lively sense, and so he goes on in the old track of staid propriety, eschewing all revivals and repeated conversions.

The religion which consists in routine and ceremony, seldom has vitality enough to become awakened to its barrenness of good works; while he whose religion consists in works and feelings is never satisfied unless he is in the whirlpool of excitement of a general revival. In like manner the conservative, respectable, reputable dentist is satisfied to be as good as the average of his fellows; but the *live*, striving, progressive dentist is never satisfied with the present attainment so long as there is an unrealized hope or an unfulfilled suggestion prophetic of greater usefulness or perfectibility of effort or purpose.

Let us now each one choose what sort of an example to set for himself and all who elect to copy, because they are too lazy to set about the profitable work under the inspiration of their own necessities!

